# Short reflex expirations (expiration reflexes) induced by mechanical stimulation of the trachea in anesthetized cats

**DOI:** 10.1186/1745-9974-4-1

**Published:** 2008-04-28

**Authors:** Ivan Poliacek, Melanie J Rose, Lu Wen-Chi Corrie, Cheng Wang, Jan Jakus, Helena Barani, Albert Stransky, Hubert Polacek, Erika Halasova, Donald C Bolser

**Affiliations:** 1 Department of Physiological Sciences, College of Veterinary Medicine, University of Florida, PO box 100144, 1600 SW Archer Road, Gainesville, Florida, 32610-0144, USA; 2 Department of Medical Biophysics, Jessenius Faculty of Medicine, Comenius University, Mala Hora 4, 037 54, Martin, Slovakia; 3 Clinic of Radiodiagnostics, Jessenius Faculty of Medicine, Comenius University, Martin, Slovakia; 4 Department of Medical Biology, Jessenius Faculty of Medicine, Comenius University, Martin, Slovakia

## Abstract

Fifty spontaneously breathing pentobarbital-anesthetized cats were used to determine the incidence rate and parameters of short reflex expirations induced by mechanical stimulation of the tracheal mucosa (ERt). The mechanical stimuli evoked coughs; in addition, 67.6% of the stimulation trials began with ERt. The expiration reflex mechanically induced from the glottis (ERg) was also analyzed (99.5% incidence, p < 0.001 compared to the incidence of ERt). We found that the amplitudes of abdominal, laryngeal abductor posterior cricoarytenoid, and laryngeal adductor thyroarytenoid electromyograms (EMG) were significantly enhanced in ERg relative to ERt. Peak intrathoracic pressure (esophageal or intra-pleural pressure) was higher during ERg than ERt. The interval between the peak in EMG activity of the posterior cricoarytenoid muscle and that of the EMG of abdominal muscles was lower in ERt compared to ERg. The duration of thyroarytenoid EMG activity associated with ERt was shorter than that in ERg. All other temporal features of the pattern of abdominal, posterior cricoarytenoid, and thyroarytenoid muscles EMGs were equivalent in ERt and ERg.

In an additional 8 cats, the effect of codeine administered via the vertebral artery was tested. Codeine, in a dose (0.03 mg/kg) that markedly suppressed cough did not significantly alter either the incidence rate or magnitudes of ERt.

In the anesthetized cat the ERt induced by mechanical stimulation of the trachea was similar to the ERg from the glottis. These two reflex responses differ substantially only in the frequency of occurrence in response to mechanical stimulus and in the intensity of motor output.

## Background

Forceful expirations are substantial part of airway defense. They arise particularly during tracheal and laryngeal coughs, sneeze, and the expiration reflex. Basic characteristics of these behaviors are known (see e.g. [1,2]) including the complex movement of the larynx [3-5].

The expiration reflex (ER) is characterized by a single and short expulsion without a preceding inspiration. ER is regularly induced from the glottis (ERg) by mechanical stimulation. Its function is to expel foreign particles from the upper airways by fast expiratory airflows [1,6]. The reflex represents a fundamental aspiration prevention mechanism [7] and is significant particularly in gastro-esophageal reflux [8], in laryngopharyngeal reflux [9,10], and under other conditions when a risk of the aspiration is markedly increased.

Several authors have observed short reflex expirations that were not preceded by an inspiration during stimulation in the trachea (ERt) of cats [11,12], dogs [13], and humans [14]. Others reported that from 1/3 [15] up to 60% ([16], also personal communication) of repetitive cough episodes induced in lower airways of anesthetized cats began with expulsion. The presence of an ER in response to mechanical stimulation of the trachea represents an important component of airway defense related to aspiration prevention. This behavior presumably is a "backup" to ER from the larynx and serves to eject foreign material from the trachea when the laryngeal ER (ERg) has failed to prevent aspiration. The extent to which these tracheal expirations (ERt) represent unique behaviors induced from stimulation of the lower airways is unknown. Some authors concluded that the ERt and ERg are the same behavior and they used the term "expiration reflex" for both of them [7,15]. However, this conclusion is based only on qualitative inspection of the motor patterns. Additional evidence is required to support the conclusion that ERt and ERg are identical reflexes.

The purpose of this study was to quantitatively examine multiple ERt induced by mechanical stimulation of the tracheobronchial airways in cats, to determine their incidence rate, and to compare their mechanical and electrophysiological characteristics with ERg. We hypothesized that ERt and ERg may represent essentially the same reflex behavior elicited from two different regions of the airways.

## Methods

All procedures were performed in accordance with the NIH Guide for the Care and Use of Laboratory Animals, the Animal Welfare Guidelines of the University of Florida, the ethical rules, and the legislation of USA and Slovakia. The protocols were approved by Ethics committee of Jessenius Faculty of Medicine Commenius University and the State veterinary administration of Slovakia (N° 5492/1999-500 and 6708/03-220) or by the University of Florida Institutional Animal Care and Use Committee (N° 8663-2004).

Experiments were performed on 58 spontaneously breathing adult cats. Fifty cats (3.44 ± 0.11 kg), 42 of them females, were used to determine an incidence rate and behavioral characteristics of the ERt. Eight cats were tested for the effect of intravertebral administration of codeine on ERt. The animals were anesthetized with sodium pentobarbital (35–40 mg/kg i.p. or i.v.). Supplementary doses were administered (1–3 mg/kg, i.v.) as needed. Atropine (0.1 – 0.2 mg/kg, i.v.) was given at the beginning of the experiment to reduce secretions. Seventeen out of 50 animals were also used in brainstem recording protocols and received hydrocortisone (9 mg/kg) to prevent brain edema. The trachea, femoral artery and vein were cannulated. An esophageal balloon was used for measuring intrathoracic pressure alterations in 33 cats and a pleural cannula was placed in 17 animals. Arterial blood pressure, end-tidal CO_2_, and body temperature were continuously monitored. Body temperature was maintained at 37.5 ± 0.5°C by a heating lamp and a pad. Arterial blood samples were periodically removed for blood gas analysis. The animals breathed air mixtures that were enriched by oxygen (25 – 60%) as required to maintain arterial pO_2 _values above 13 kPa (100 mm Hg).

In 8 cats a cannula was introduced into the left brachial artery and the tip was positioned near the origin of the vertebral artery. All other branches of the subclavian artery in the region were clamped so the codeine (a single dose of 0.03 mg/kg) was delivered directly to the brainstem circulation.

Electromyograms (EMG) of respiratory muscles were recorded with bipolar insulated fine wire electrodes. We recorded EMGs from the expiratory abdominal muscles (ABD) transversus abdominis, rectus abdominis or external oblique, from the inspiratory parasternal muscles (PS), in 17 animals and in an additional 8 "codeine" cats alternatively from the diaphragm (DIA), in 42 animals from the inspiratory laryngeal posterior cricoarytoneid muscle (PCA), and in 39 cats from the expiratory laryngeal thyroarytenoid muscle (ThAr). The PS electrodes were placed at T3 proximal to the sternum. The DIA electrodes were introduced into the crural diaphragm. Transversus abdominis and rectus abdominis (or external oblique) electrodes were placed 3–4, respectively 1 cm lateral to the linea alba. The PCA electrodes were inserted along the dorsal surface of the arytenoid cartilage using its dorsal edge as a visual cue after gently elevating the larynx. The ThAr electrodes were inserted directly through the cricothyroid membrane. Proper placement of each set of electrodes was confirmed by the appropriate inspiratory or expiratory phased activity during breathing and other respiratory events as well as by visual inspection.

Animals (except the 8 codeine cats) were placed prone in a stereotaxic frame and the dorsal surface of medulla was exposed by an occipital craniotomy for later interventions in the brainstem under another protocol. The medullary surface was covered by warm paraffin oil.

**Figure 1:**
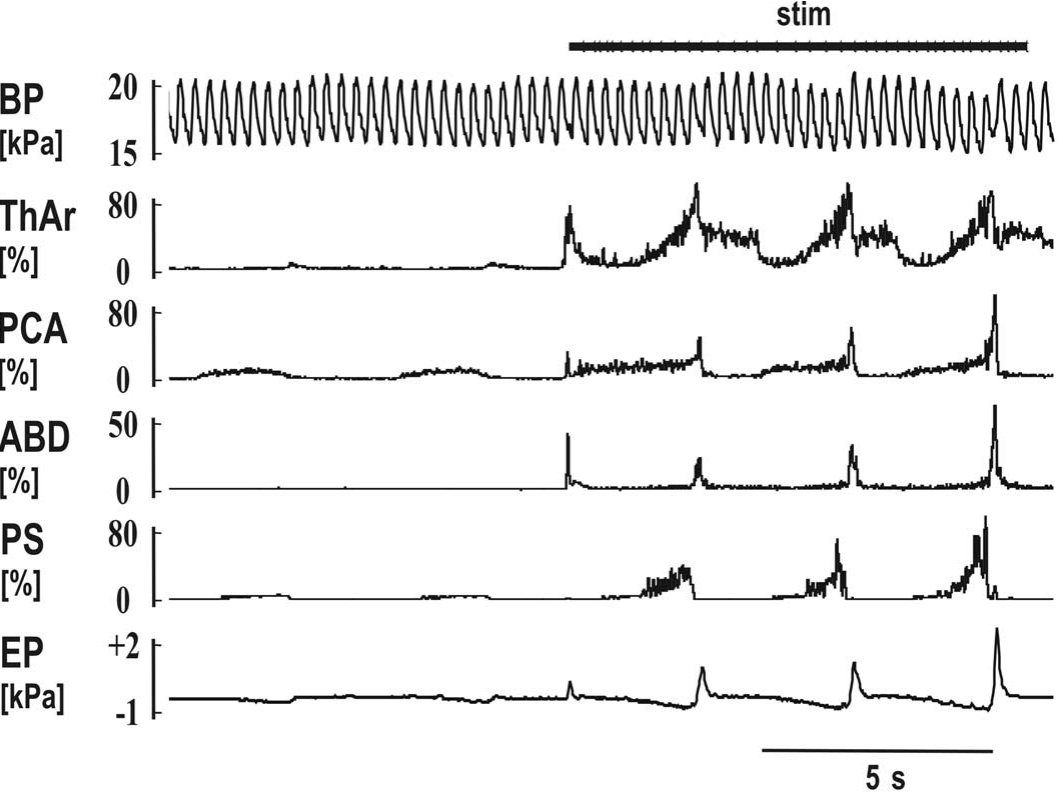
**The reflex responses to the mechanical stimulation (stim) in the trachea**. Two quiet breaths (slight inspiratory increase in the records of laryngeal abductor posterior cricoarytenoid – PCA and parasternal muscles – PS EMG moving averages with a depression in esophageal pressure – EP) followed by a short reflex expiration (ERt) at the beginning of stimulation (steep elevations in EMG moving averages of laryngeal adductor thyroarytenoid muscle – ThAr, PCA, and abdominal muscles – ABD, as well as in EP). The ERt was then followed by 3 coughs in which the expulsions caused slight elevations of blood pressure (BP). Moderate post-inspiratory activity was present at the inspiratory-expiratory transition of quiet breathing in ThAr. The ERt was markedly shorter compared to the cough expulsions (see ABD and EP) leading to the lower amplitude of EP compared to that in coughs, although the amplitudes of ABD EMG moving averages remained comparable.

Mechanical stimulation of the intrathoracic trachea (between the edge of tracheal cannula and the carina) was performed with a thin polyethylene catheter (diameter 0.5 – 1.0 mm) or nylon fiber (diameter 0.2 – 0.5 mm) for the period of 5–20 s. Six to 18 stimulation trials (11.2 trials in an average) were conducted without any additional intervention (the time interval between stimulation trials was approximately 1 minute). The stimulations elicited ERt and single or repetitive coughs (Fig. 1). We used a mechanical stimulus on the glottis with the thin nylon fiber (diameter 0.2 mm) in order to induce ERg. In an additional 8 codeine cats, 20 – 30 mechanical stimulation trials were conducted to establish a stable cough baseline. Then 5 control pre-codeine stimulus trials were applied during the period of 5 min, followed by 5 stimulus trials after the intra-vertebral administration of the codeine (0.03 mg/kg).

**Figure 2:**
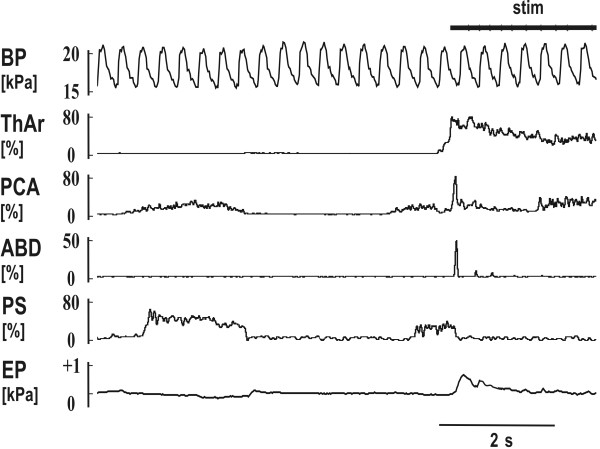
**Mechanical stimulation of the trachea (stim) with short reflex expiration (ERt) during the inspiratory period of breathing**. The stimulation immediately terminated an inspiration (rapid suppression of PCA and PS) and induced the ERt (abrupt activation of ThAr, PCA and ABD). Two much weaker ERt were detectable in the record of ABD, before the initial cough inspiration began (activation of PCA at the end of the record). See Fig. 1 for abbreviations.

**Figure 3:**
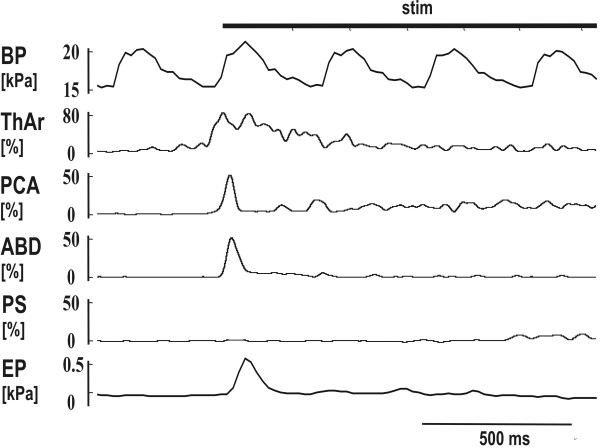
**Motor pattern of short reflex expiration (ERt) induced by mechanical stimulation of the trachea (stim)**. See Fig. 1 for abbreviations.

The ERt from the trachea (Fig. 1, 2 and 3) and ERg from the glottis were both defined as a brief short burst of ABD electrical activity with positive swing of esophageal or pleural pressure without a preceding inspiration. The response induced in the inspiratory phase of breathing regularly and immediately terminated inspiration. No coactivation of inspiratory (PS or DIA) and expiratory activity (ABD) was observed either in ERt (Fig. 2) or in ERg [4,5]. Cough was defined as a coordinated inspiratory-expiratory sequence manifest as a large burst of inspiratory EMG activity immediately followed by a burst of expiratory ABD activity with an inspiratory-expiratory waveform of intrathoracic pressure (Fig. 1). These criteria separated ERt or ERg from cough and also from other airway defensive behaviors such as augmented breath and aspiration reflex.

All EMGs were amplified, filtered (300 – 5000 Hz), rectified, and integrated (time constant 50 ms). We analyzed for both the ERt and ERg: (1) the number and the incidence rate, (2) the amplitudes of ABD, PCA and ThAr EMG moving averages, (3) the peak of esophageal or pleural pressure, (4) the duration and time correlations of PCA, ThAr, and ABD activities. Magnitudes of the ABD, PCA, and ThAr moving averages were normalized to the strongest tracheal cough reflex. The characteristics of ERt and ERg in cats treated with hydrocortisone and chest wall surgery (in order to insert pleural cannula, 17 cats) were similar to those measurements taken from animals without such interventions (33 cats). Thus, the final analysis was performed on all these 50 cats.

Results are expressed as a mean values ± SEM. Incidence rate of ERt and ERg, their occurrence in trials that began during inspiration and expiration, the number of cats selected for analysis of ERt/ERg parameters (ERt or ERg ≥ 0.2 kPa (> 1.5 mm Hg)), and the number of animals with analyzed laryngeal muscles EMGs were processed with the Fisher's exact test. The parameters of ERt and ERg were compared using unpaired t-test, Welch corrected unpaired t-test, and Mann-Whitney test (Table 1). The paired t-test was used to compare ERt ABD EMG amplitudes in codeine-treated cats. The differences of variables were considered significant at p < 0.05.

**Table 1:** The parameters of short reflex expirations induced by mechanical stimulation of the trachea (ERt) and expiration reflexes from the glottis (ERg).

	**ERt**	**ERg**	p (test)
Number of cats	50	31	
Excitability (responses/trials)	67.6%	99.5%	< 0.001 (F)

Cats with several ERt/ERg ≥ 0.2 kPa	28 (out of 50)	27 (out of 31)	< 0.01 (F)
Duration of ABD	63 ± 3 ms	70 ± 3 ms	NS (t)
T (onset of ABD – ABD peak)	36 ± 3 ms	39 ± 2 ms	NS (t)
Relative amplitude of ABD	36 ± 4%	60 ± 9%	< 0.02 (W)
Expiatory pressure amplitude	0.49 ± 0.04 kPa	1.02 ± 0.16 kPa	< 0.01 (W)

Number of cats – PCA	23	22	NS (F)
T (onset of PCA – ABD peak)	52 ± 3 ms	61 ± 4 ms	NS (t)
T (peak of PCA – ABD peak)	14 ± 2 ms	25 ± 4 ms	< 0.02 (W)
T (ABD peak – offset of PCA)	24 ± 3 ms	18 ± 6 ms	NS (W)
Relative amplitude of PCA	67 ± 12%	137 ± 23%	< 0.001 (M)

Number of cats – ThAr	20	22	NS (F)
T (onset of ThAr – ABD peak)	77 ± 4 ms	74 ± 6 ms	NS (W)
T (peak of ThAr – ABD peak)	44 ± 3 ms	43 ± 3 ms	NS (t)
T (ABD peak – minimum of ThAr)	7 ± 4 ms	21 ± 8 ms	NS (W)
T (ABD peak – 2^nd ^peak of ThAr)	140 ± 20 ms	130 ± 10 ms	NS (W)
T (ABD peak – offset of ThAr)	440 ± 40 ms	730 ± 60 ms	< 0.001 (W)
Relative amplitude of 1^st ^ThAr peak	83 ± 10%	385 ± 77%	< 0.001 (W)
Relative amplitude of 2^nd ^ThAr peak	85 ± 20%	311 ± 70%	< 0.01 (W)

ABD, PCA, and ThAr, EMG activities of abdominal, posterior cricoarytenoid, and thyroarytenoid muscles during ERt or ERg; T (onset of ABD – ABD peak), the time interval from the beginning of the burst of ABD to the moment when the maximum of the moving average was reached (amplitude of ABD moving average); other time intervals (T) were measured as described in the table; p (test), the level of statistical significance and in brackets the appropriate test used; NS, non-significant (p > 0.05); F, Fisher's test; t, unpaired t-test; W, Welch corrected unpaired t-test; M, Mann-Whitney test.

## Results

We conducted 562 tracheal stimulation trials in 50 animals, 326 of them began in the expiratory period of breathing (58%), 236 in inspiration (42%). The stimulation induced cough (Fig. 1) and during 380 stimulation trials also ERt (67.6%, Fig. 1, 2 and 3). ERt typically appeared at the beginning or very early stage of the tracheal stimulation (Fig. 1, 2 and 3). For 380 stimulation trials with ERt, 263 trials began in expiration (69.2%), 117 in inspiration (30.8%). No ERt were detected in 182 trials (32.4%); 117 of these non-responding stimulations began during inspiration (64.3%,), 65 trials in expiration (35.7%). ERt was significantly more elicitable in expiration (p < 0.001, Fisher's test).

We selected 28 animals with multiple ERt in which the magnitude of the expulsion reached at least 0.2 kPa (120 ERt, 99 of them induced in expiration, 21 in inspiration) for further analysis. The ABD EMG of 34 out of 120 ERt consisted of two bursts in close succession; another 26 were multiple burst complexes. Only the largest component of these multi-burst responses was measured. The characteristics of the PCA EMG were examined during 96 ERt in 23 cats and that of ThAr during 86 ERt in 20 cats (Table 1). The PCA EMG responded with a short burst of activity that slightly preceded the ABD activity. The ThAr was activated even earlier than the PCA and then suppressed while ABD EMG activity reached its peak (Fig. 3). However, a prolongation of ThAr activity during ERt was recorded in 62 out of 86 ERt; a second prolonged activity of ThAr appeared after the ABD burst (Table 1, Fig. 1, 2 and 3).

Glottal stimuli applied in 31 animals produced ERg 99.5% of the time (426 out of 428 stimulations, 279 during expiration and 149 during inspiration). The animals with multiple ERg, pressure amplitudes of which reached at least 0.2 kPa, were included in further analysis (211 ERg in 27 cats, 150 of them in expiration and 61 in inspiration; Table 1). The features of laryngeal muscle activities were measured on 176 ERg in 22 cats (Table 1).

No significant differences between the characteristics of ERt or ERg that were induced in expiration vs. inspiration were found.

The intra-vertebral administration of codeine (0.03 mg/kg) did not affect the incidence rate of ERt (23/40 vs. 21/40 in control, p > 0.81, Fisher's test) and their ABD EMG amplitudes (5 ± 1% vs. 10 ± 4% in control, p > 0.32, paired t-test) compared to the ERt in pre-codeine control. This intervention reduced the number of tracheal coughs by 73% (p < 0.01, paired t-test) and cough ABD EMG amplitudes from 46 ± 8% to 9 ± 4% (p < 0.01, paired t-test).

## Discussion

The major finding of this study was that quantitative analysis of mechanically induced ERt and ERg revealed a high degree of similarity between these two behaviors.

The patterns of ABD, PCA, and ThAr were similar during both ERt and ERg. Laryngeal adductor ThAr was activated first, then PCA and ABD followed. During the maximum ABD bursting suppression of ThAr was detected that was frequently followed by another prolonged burst of ThAr activity. We propose that such patterns in activation of ThAr, PCA, and ABD may represent 3 phases of laryngeal movement during ERt, which are the *compressive, expulsive, and subsequent constriction phase *analogously to those found in ERg [4,5,17].

We found higher amplitudes of ABD, PCA, and ThAr EMG moving averages, as well as higher pressure amplitudes in ERg than those in ERt. The larynx is considered a very sensitive area with a high density of receptors [18,19]. Laryngeal abductors and adductors were more vigorously activated during the ERg and cough from the larynx, compared to cough from the tracheal region [4]. As such, stronger activation of laryngeal and abdominal motor outputs might arise with stimulation of the larynx. Distinct sites of stimulation (glottis vs. trachea) could also account for the longer duration of ThAr activity and the earlier PCA maximum in ERg than in ERt (Table 1).

The frequency of occurrence of ERt was significantly lower (67.6%) in our tracheal stimulation trials than that of ERg. Tomori and Stransky [12] mechanically induced expiratory responses from the glottis, subglottal, tracheal, and nasal mucosa in anesthetized cats. They also saw a higher incidence rate of ERg (85%) than that of ERt (50%). However, the pressure amplitudes were higher in their study compared with our results, particularly in the case of ERt (1.2 kPa vs. our 0.49 kPa). The experimental preparations could account for the differences in results between the two studies, such as craniotomy on our cats and different patterns of stimulation (brief tactile tracheal stimuli vs. the continuous stimulation in present experiments). Tatar et al. [15] also indicated a more frequent occurrence of ERg compared to ERt in cats and rabbits.

The ERt appeared more frequently at the beginning of stimulations that started during the expiratory period of breathing (see results). This is in line with vigorous expression of ERg when stimuli were delivered during expiration, particularly at the beginning of an expiratory period [20,21]. We also saw a higher incidence rate of ERg with pressure amplitudes at least 0.2 kPa in expiration than those in inspiration (p < 0.02, Fisher's test). However, we did not find any significant differences in the parameters of ERt (and also ERg) that were induced during inspiration vs. those ERt (ERg) in expiration. We have to point out that the analyzed ERt (ERg) in inspiration were the strongest responses obtained in the phase (assuming the criterion of at least 0.2 kPa of pressure amplitude).

ERt, as well as the ERg, differ substantially from cough. As we stated in the results, ERt appeared as a single (Fig. 1) or sometimes as a few bursts (Fig. 2) just at the beginning of tracheal stimulation. The response was induced presumably due to an immediate contact of the stimulation device with the tracheal mucosa and before the threshold for the cough response was achieved. ERg can occur repeatedly in response to mechanical stimulation of the larynx [1], but ERt has never been reported to occur repetitively in response to mechanical stimulation of the tracheal-bronchial region [7]. Vovk et al. [22] also found a low incidence rate of repeated ER in humans exposed to irritant aerosols, particularly when compared with the number of coughs. We did not quantify the number of coughs in our stimulation trials. However, the cough number was typically higher than the number of ERt (Fig. 1). It is commonly accepted that cough is the primary (and most frequent) response from the tracheal area and that the ER is preferentially (and more frequently) induced from the larynx [1,5,7]. ERt in our study were never detected after the beginning of the initial cough inspiration. This observation also suggests that the ERt has a shorter latency for onset relative to coughing. The latency of cough response is typically several hundred ms [23,24], for ERg it is about 30 ms [12,25]. The laryngeal muscles are activated even earlier (Table 1), which corresponds to the very short latencies of responses detected in laryngeal motor output following the superior laryngeal nerve stimulation [26,27].

The motor pattern as well as a function of cough and ER differ significantly [1,5,7]. Cough is an inspiratory-expiratory behavior (Fig. 1) [1,5,7]. EMG activities of inspiratory and expiratory pump muscles were coactivated during the inspiratory phase of cough and at the inspiratory-expiratory transition (pre-expulsive ABD activity) [28]. However, similar to ERg [17,20], when we induced the ERt in the inspiratory period of breathing the inspiration was immediately terminated and the expiratory response followed after a short delay (Fig. 2). The ABD activity in cough is substantially longer and stronger (Table 1; Fig. 1) than that in ERt or ERg (see also [1,4,12]). Shorter expulsive phase durations and lower airflows of ER than those of cough expulsions were reported after capsaicin challenge on humans [22]. We documented that the activation of the laryngeal muscles is also prolonged during cough relative the ERg [4]. Moreover, there is a sequential and biphasic activation and inhibition of laryngeal abductors and adductors in cough [4,5,29]. During ERt (Fig. 1, 2 and 3) or ERg [4,5], only the adductor activation presents as a biphasic response; the PCA is activated in a single short burst.

The ERt and ERg share other important characteristics distinct from those of coughing [7,15]. ERt/ERg depend on lung capacity, particularly when they occur during the expiratory period of the respiratory cycle. As such, there is strong positive correlation between lung inflation and pressure amplitudes of ER [30-32]. No such clear relationship was found for cough [31,32]. The effects of general anesthesia on cough and ERt/ERg also differ substantially. Increased anesthetic levels have a more pronounced suppressive impact on tracheal cough than on the ER [1,11]. The proportion of ERt to coughs was increased in anesthetized cats compared with awake animals [15]. Although the authors did not specifically identify ERt in their paper, inspection of May and Widdicombe [33] data suggests that morphine inhibited cough more than mechanically induced ERt. Similar findings were reported for ERg after the administration of codeine [1]. However, the effect of codeine on ERt has not been reported previously. We found that the codeine, a potent cough inhibitor in anesthetized cats [34], had little suppressive effect on ERt. As such, there is no difference in the response of ERt and ERg to central antitussives and this finding differs markedly from the response of cough to these drugs.

Vovk et al. [22] analyzed cough and ER induced by capsaicin challenge in healthy awake humans. The authors distinguished repetitive ER and repetitive cough expulsions (successive cough expirations), which were associated with a single initial cough inspiration and termed this phenomenon re-acceleration of expulsive airflow. Widdicombe and Fontana [7] discussed frequent "cough" patterns containing individual and repetitive coughs (with single or multiple expulsions) and eventually ERt/ERg. Multiple (divided) expulsions with intermittent flow might create more turbulent airflow and thus more efficient shear forces along the walls of the lower airways than the continuous airflow of a single expiration. The rate of occurrence of such multiple cough expulsions in cough animal models, particularly in the cat, is unknown; however, this number is low in accord with our own as well as the observations of others [1]. Because subsequent cough expulsions are consistent with the definition of ER, the nomenclature of ER (also in accord with [7,22]) should be re-examined. We propose to characterize the ER as a family of behaviors (ERt and ERg) that: a) are short in duration (typically shorter compared to cough expulsion), and b) nonrhythmic (without cyclic – rhythmic feature typical for cough) [35] expiratory responses consisting of compression and expulsion, which are not dependent on a preceding inspiration. The ERt/ERg are likely produced and controlled by different mechanisms than the expulsions in cough [5,7,22,35]. Distinct neuronal circuitries generating the central motor patterns of the cough [36] and ER [37] have already been proposed.

## Conclusion

Our quantitative analyses as well as the reports of others on the ERt and ERg vs. cough suggest that (1) ERt should be considered a different reflex response from cough and (2) ERt and ERg may represent the same defensive reflex, which has a different frequency of occurrence and intensity when induced from two distinct areas of the airways.

## List of abbreviations

ABD: Abdominal muscles; DIA: Diaphragm; EMG: Electromyogram (electromyographic); ER: Expiration reflex; ERg: Expiration reflex from the glottis or larynx; ERt: Short reflex expiration (ER from the trachea); PCA: Posterior cricoarytenoid muscle; PS: Parasternal muscles; ThAr: Thyroarytenoid muscle.

## Competing interests

The authors declare that they have no competing interests.

## Authors' contributions

All authors have met criteria for an authorship of the article. IP assembled the study, participated on the experimental design, carried out experiments, analyzed data, and drafted the manuscript; MJR carried out experiments, contributed to the recording and processing of data, and analyzed "codeine" data; LWChC carried out experiments, contributed to the final analysis of data and to the manuscript; ChW performed experiments and contributed to the recording and processing procedures; JJ designed and performed experiments, contributed to the manuscript; HB performed experiments and carried out recording and processing of data; AS participated on experimental design and on the draft of manuscript; HP participated on experimental design and data analysis; EH performed experiments and contributed to the manuscript; DCB designed and coordinated experiments, participated on final analysis and on the draft of the manuscript. All authors have read and approved the final manuscript.
